# Comparison of perioperative, oncologic, and functional outcomes between 3D and 2D laparoscopic radical prostatectomy: a systemic review and meta-analysis

**DOI:** 10.3389/fonc.2023.1249683

**Published:** 2023-09-19

**Authors:** Hui Shuai, Xi Duan, Tao Wu

**Affiliations:** ^1^ Department of Urology, Affiliated Hospital of North Sichuan Medical College, Nanchong, Sichuan, China; ^2^ Department of Dermatology, Affiliated Hospital of North Sichuan Medical College, Nanchong, Sichuan, China

**Keywords:** prostate cancer, radical prostatectomy, 3D laparoscopy, technology, meta-analysis

## Abstract

**Objectives:**

Literature regarding experience with 3D laparoscopy about prostatectomy has remained scanty, and this could be related to the rise of robotic assisted laparoscopic surgery. This study aimed to perform a systemic review and meta-analysis to evaluate the perioperative, functional, and oncologic outcomes between 3D and 2D laparoscopic radical prostatectomy (LRP).

**Methods:**

We systematically searched the PubMed, Embase, and Cochrane Library databases for studies that compared perioperative, functional, or oncologic outcomes of both 3D and 2D LRP. The Newcastle-Ottawa Scale (NOS) tool and Jadad scale were used to assess the risk of bias in the included studies. Review Manager 5.3 was used for the meta-analysis.

**Results:**

Seven studies with a total of 542 patients were included in the analysis. Among them, two were RCTs. There was no difference between groups in terms of preoperative characteristics. Anastomosis time, hospital day, and overall complication rates were similar in 3D than 2D group. However, operative time [mean difference (MD) -36.96; 95% confidence interval [CI] -59.25 to -14.67; p = 0.001], blood loss (MD -83.5; 95% CI -123.05 to -43.94; p <0.0001), and days of drainage (MD -1.48; 95% CI -2.29 to -0.67; p = 0.0003) were lower in 3D LRP. 2D and 3D LRP showed similarity in the positive surgical margin (PSM) rate and biochemical recurrence (BCR) rate at 3, 6, and 12months postoperatively. Additionally, there was no significant differences in continence and potency recovery rate between two group except higher continence rate of 3D LRP at 3 months.

**Conclusion:**

Current evidence shows that 3D LRP offers favorable outcomes compared with 2D LRP, including operative time, blood loss, days of drainage, and early continence. However, there was no conclusive evidence that 3D LRP was advantaged in terms of oncologic and functional outcomes (except for continence rate at 3 months).

**Systematic review registration:**

The study has been registered on the International Prospective Register of Systematic Reviews (PROSPERO: CRD42023426403).

## Introduction

Prostate cancer (PCa) is the most commonly diagnosed cancer except lung cancer and the fifth leading cause of cancer death in males around the world, accounting for 14.1% (1,414,259) of total new cancer cases and 6.8% (375,304) of total cancer deaths in males in 2020 (1, 2). Since Schuessler first conducted laparoscopic radical prostatectomy (LRP) in 1992 (3), LRP has become a standard treatment for organ confined prostate cancer (4). However, widespread application of LRP has been restricted by some limitations, including two-dimensional (2D) vision, limited degrees of freedom, lacked stereoscopic perception, and a steep learning curve.

In this context, three-dimensional (3D) laparoscopic surgery is gradually emerging. The development of 3D laparoscopic technology attempts to overcome the 2D vision of traditional laparoscopic surgery. Previously, 3D systems made surgeons more prone to fatigue and harmful to the eyes (2, 5). These limitations hampered the widespread application of 3D laparoscopic technology in clinics. With the improvement of surgical navigation and augmented reality, 3D laparoscopic technology has taken an important step towards clinical practice (6). New-generation 3D laparoscopic technology had high-definition stable images, increasing the comfort of glasses and reducing the fatigue of surgeons (7) Nowadays, 3D laparoscopy is becoming increasingly popular worldwide.

On the other side, literature regarding experience with 3D laparoscopy about prostatectomy has remained scanty, and this could be related to the rise of robotic assisted laparoscopic surgery.

This study aimed to perform a systemic review and meta-analysis to evaluate the perioperative, functional, and oncologic outcomes between 3D and 2D LRP.

## Methods

The systematic review and meta-analysis were based on the Preferred Reporting Items for Systematic Reviews and Meta-Analyses (PRISMA) statement (8).

### Eligibility criteria

Eligibility criteria were formulated using the specific population, intervention, comparison, outcomes, and study design (PICOS) framework. This review included studies that met the following criteria: (P): patients with organ confined PCa; (I): undergoing 3D LRP; (C): in which traditional 2D LRP was performed as comparator; (O): perioperative, oncologic, and functional outcomes; and (S): retrospective and prospective cohort studies.

Case series, surveys, letters, editorial comments, reviews, and animal studies were not included. In addition, studies without original data and articles in languages other than English were excluded.

### Information sources, search strategy, and selection process

A systematic search was conducted using Embase, PubMed, and the Cochrane Library. The search terms used were: (Prostatectomy OR Prostatectomies OR Prostatectomy, Suprapubic OR Prostatectomies, Suprapubic OR Suprapubic Prostatectomies OR Suprapubic Prostatectomy OR Prostatectomy, Retropubic OR Prostatectomies, Retropubic OR Retropubic Prostatectomies OR Retropubic Prostatectomy) AND 3D.

The search results were limited to humans. Studies published between January 1, 2010, and April 1, 2023, were included. Articles were reviewed by two authors (S.H and D.X) according to *a priori* inclusion/exclusion criteria. Any conflicts about eligibility were resolved between the two authors and any disagreements were resolved by a third party (W.T). Studies that meet our PICOS criteria were included.

### Data collection process and data items

The authors extracted data from the seven included studies. Data extracted included study characteristics (first author, year of publication, country, study design, number of participants), baseline demographics [age, body mass index (BMI), and prostate-specific antigen (PSA)], perioperative (operative time, anastomosis time, number anastomosis stitches, blood loss, hospital day, days of drainage/catheterization, complications), oncologic (PSM, BCR-free), and functional (urinary continence and potency) outcomes.

### Risk of bias assessment

The Newcastle–Ottawa Scale (NOS) (9, 10) was used to assess the quality of non-RCT. The NOS checklist includes three quality parameters: population selection (4 points), comparability of cohorts (2 points), and assessment of outcome for cohort studies (3 points). Each study received a score ranging from 0 to 9. Studies with a score of 7 or higher were considered high-quality articles. And a risk of bias assessment was conducted on RCTs using the Jadad scale (11). A total score of 1–2 was considered low quality and 3–5 was considered high quality.

### Synthesis methods

The meta-analysis included retrospective and prospective cohort studies and was performed using Review Manager 5.3 (Cochrane Collaboration, Oxford, UK). We pooled clinical effect estimates using the mean difference (MD), relative risk (RR), and their respective 95% CIs. The statistical significance level was set at *p* < 0.05. The Mantel–Haenszel effects model and inverse-variance effects model were used to combine the trials. We calculated and depicted forest plots with a 95% CI. The *I^2^* test and Cochran’s Q test were used to assess the heterogeneity. Statistical heterogeneity was indicated by *p* < 0.1 in the Cochran’s Q test and *I^2^* > 50% in the I^2^ test. If heterogeneity existed, a random effect model was adopted; otherwise, a fixed effect model was adopted. *I^2^* values of 25%, 50%, and 75% indicate low, moderate, and high levels of inconsistency, respectively (12). Further sensitivity analyses were conducted to reduce heterogeneity and confirm the reliability of our findings.

## Results

### Study selection, characteristics, and risk of bias

**Figure 1:**
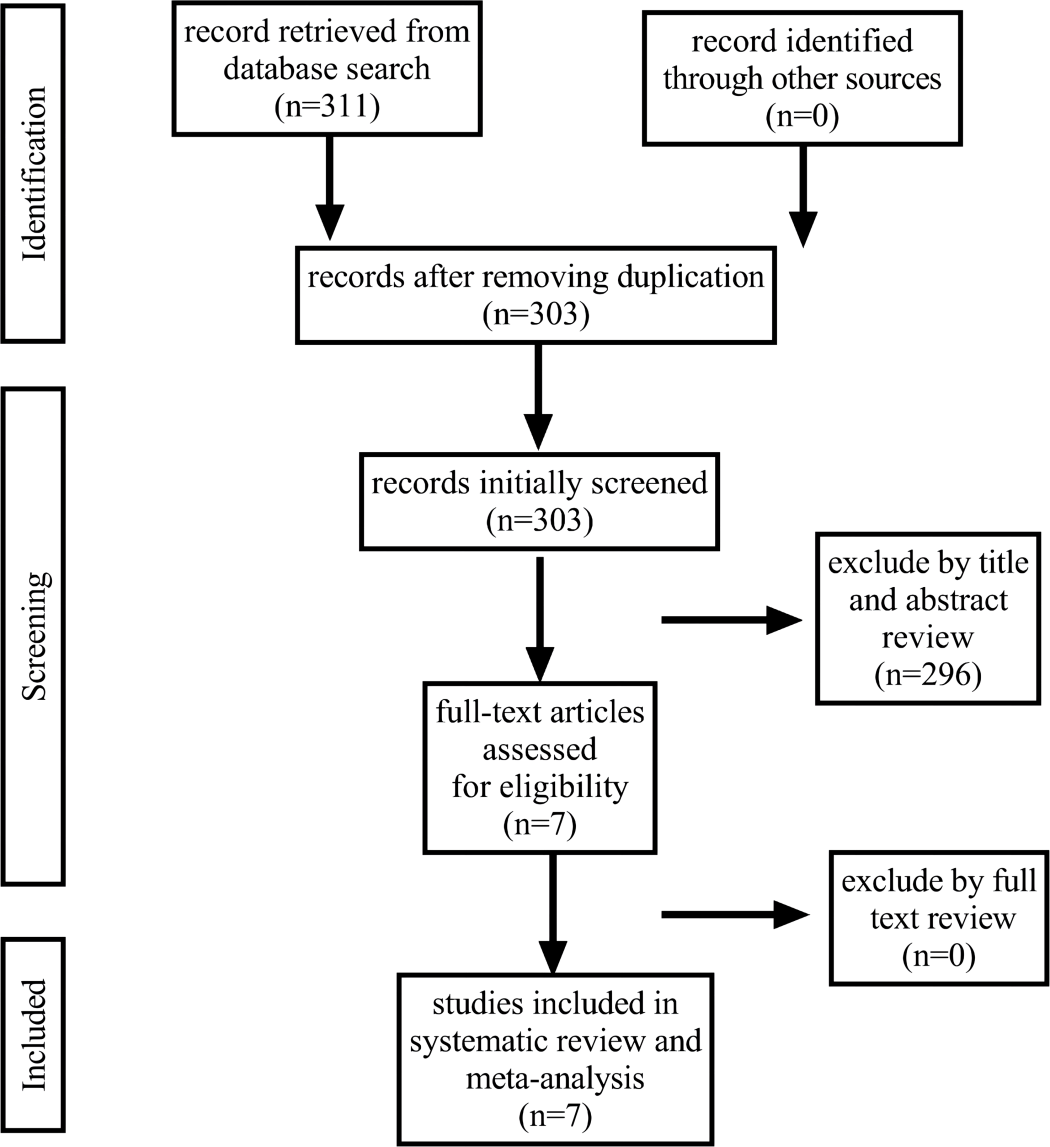
Flowchart illustrating the major steps of the review process in accordance with the Preferred Reporting Items for Systematic Reviews and Meta-analyses (PRISMA) statement.

Our initial research identified 311 articles, of which 7 were selected for further analysis. **Figure 1** depicts the search process (PRISMA flowchart). Three studies were prospective (13–15), and four were retrospective (16–19). Two of them were RCTs (13, 15). Among the 542 patients included in the meta-analysis, 300 (55.4%) and 242 (44.6%) were 2D and 3D LRP, respectively. Among 7 articles included, all studies reported results on perioperative outcomes and complications (13–19), 4 studies on urinary continence (14–16, 19), 3 studies on potency (14, 15, 19), 4 studies on PSM (14–16, 19), and 3 articles on BCR (14, 15, 19). **Table 1** provides an overview of the patients and details of our study population. All the included studies are of high quality.

**Table 1:** Characteristics of the included studies.

Author	Year	Country	design	No.patients	Age, years	BMI, kg/m^2^	PSA, ng/ml	Quality
Kinoshita (13)	2014	Japan	Prospective	2D 57 3D 59	65.9 ± 4.7 66.5 ± 4.5	23.6 ± 2.5 23.7 ± 2.5	8.7 ± 6.4 7.2 ± 5.0	4 (RCT)
Aykan (16)	2014	Turkey	Retrospective	2D 72 3D 29	64.5 (46–78) 65 (49–73)	30.5 (24–37) 31 (25–35)	6.0 (2.3–34.7) 7.6 (4.0–23.0)	7
Kyriazis (17)	2015	Greece	Retrospective	2D 10 3D 5	–	–	–	8
Bove (14)	2015	Italy	Prospective	2D 43 3D 43	60.1 63.9	25.2 24.6	6.7 6.2	8
Bin (18)	2015	China	Retrospective	2D 32 3D 18	67.8 ± 8.4 67.3 ± 6.6	24.3 ± 3.9 24.1 ± 4.3	10.2 ± 4.3 9.8 ± 5.8	7
Kaiqiang (19)	2017	China	Retrospective	2D 36 3D 36	66.03 ± 5.74 65.14 ± 9.15	24 ± 2.30 23.91 ± 3.10	12.02 ± 10.58 12.09 ± 10.22	7
Benelli (15)	2018	Italy	Prospective	2D 50 3D 52	58.2 60.5	25.4 24.7	7.4 6.8	4 (RCT)

2D two-dimensional, 3D three-dimensional, BMI body mass index, PSA prostate-specific antigen, and RCT randomized controlled trial.

### Perioperative outcomes and complications

**Table 2** presents the results of operative time, anastomosis time, number anastomosis stitches, blood loss, hospital stay, days of drainage/catheterization, and overall complications from the studies comparing 2D and 3D LRP.

**Table 2:** Perioperative outcomes and complications of the included studies.

Study	Surgical technique	Operative time (min)	Anastomosis time (min)	Number anastomosis stitches	Blood loss (ml)	Hospital day	Days of drainage	Days of catheterization	Complications
Kinoshita (13)	2D	148 ± 43	30.1 ± 13.5	11.5 ± 3.2	–	–	–	–	–
	3D	150 ± 53	26.7 ± 8.7	10.4 ± 2.1					
Aykan (16)	2D	190 ± 31	87 ± 17	–	138 ± 32	–	–	–	3/72
	3D	131 ± 18	28 ± 6		102 ± 17				0/29
Kyriazis (17)	2D	71.5 ± 7.65	–	–	–	1.4 ± 0.26	–	–	–
	3D	80.04 ± 12.5				2.4 ± 1.02			
Bove (14)	2D	241 ± 51	32 ± 6.4	6.45	532 ± 459	7.6	4.5	10.55	13/43
	3D	162 ± 18	24 ± 8.9	5.65	383 ± 102	5.5	4.85	10.75	8/43
Bin (18)	2D	180.2 ± 69.1	–	–	236.5 ± 60.6	20.2 ± 5.5	7.1 ± 1.1	–	1/32
	3D	118.3 ± 55.1			89.1 ± 35.2	14.4 ± 7.2	5.3 ± 2.1		0/18
Kaiqiang (19)	2D	218.11 ± 35.96	–	–	177.8 ± 102.4	12 ± 5.23	7.33 ± 3.20	25.39 ± 6.88	5/36
	3D	167.72 ± 26.42			86.1 ± 57.8	11.47 ± 4.93	6.33 ± 2.29	25 ± 6.78	4/36
Benelli (15)	2D	143 ± 17	31 ± 12	–	230 ± 30	7.8	2.8	9.6	6/50
	3D	118 ± 15	23 ± 12		180 ± 40	6.1	3.1	8.2	8/52

**Figure 2:**
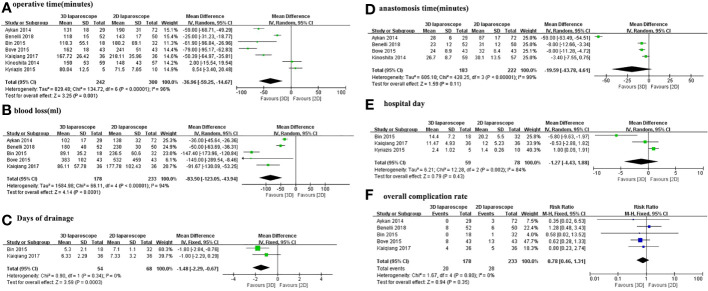
Forest plot comparing the perioperative outcomes of 3D and 2D LRP. **(A)** Operative time; **(B)** Anastomosis time; **(C)** Blood loss; **(D)** Hospital day; **(E)** Days of drainage; **(F)** Overall complication rate.

**Figure 2** depicts the pooled results of overall perioperative outcomes and complications. Meta-analysis demonstrates that the pooled estimates of operative time (MD -83.5; 95% CI -123.05 to -43.94; *p <*0.0001), blood loss (MD -83.5; 95%CI -123.05 to -43.94; *p <*0.0001), and days of drainage (MD -1.48; 95% CI -2.29 to -0.67; *p* = 0.0003) were lower in 3D LRP than those in 2D LRP (**Figures 2A-C**). No statistically significant differences were found in terms of anastomosis time, hospital day (**Figures 2D, E**).

Moreover, the overall complication rates were 11.2% (20 out of 178 cases) for 3D LRP and 12% (28 of 233 cases) for 2D LRP, respectively. Pooled results from five studies showed no significant differences in the overall complication rates (RR 0.78; 95% CI 0.46 to 1.31; *p*=0.35) (**Figure 2F**).

### Oncological outcomes

**Figure 3:**
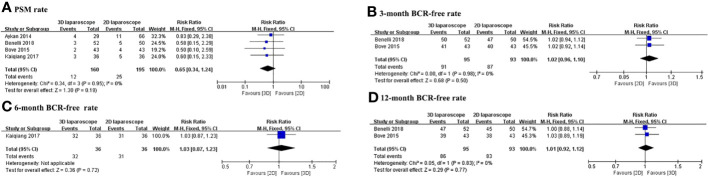
Forest plot comparing the oncologic outcomes of 3D and 2D LRP. **(A)** Positive surgical margin rate; BCR-free rate at 3 **(B)**, 6 **(C)**, and 12 **(D)** months.

The PSM and BCR-free rates for 3D and 2D LRP are presented in **Table 3**. **Figure 3** depicts the pooled results of oncological outcomes. Meta-analysis demonstrates that there were no statistically significant differences between 3D and 2D LRP group concerning PSM and BCR-free at 3, 6, and 12months (**Figure 3**). Two studies used PSA>0.2 ng/ml to define BCR (14, 19) and two studies did not define the cutoff used (15, 16).

**Table 3:** Oncologic outcomes of the included studies.

Study	Surgical technique	Histopathologic data, n(%)		PSM	BCR definition (ng/ml)	BCR-free		
		≤ p2	≥ p3			3 mo	6 mo	12 mo
Aykan (16)	2D	51(77%)	15(23%)	11/66	–	–	–	–
	3D	24(83%)	5(17%)	4/29				
Bove (14)	2D	33(77%)	10(23%)	4/43	PSA>0.2	40/43	–	38/43
	3D	32(75%)	11(25%)	2/43		41/43		39/43
Kaiqiang (19)	2D	33(92%)	3(8%)	5/36	PSA>0.2	–	31/36	–
	3D	32(89%)	4(11%)	3/36			32/36	
Benelli (15)	2D	36(72%)	14(28%)	5/50	–	47/50	–	45/50
	3D	38(76%)	12(24%)	3/52		50/52		47/52

PSM positive surgical margin, BCR biochemical recurrence, PSA prostate-specific antigen.

### Functional outcomes

**Figure 4:**
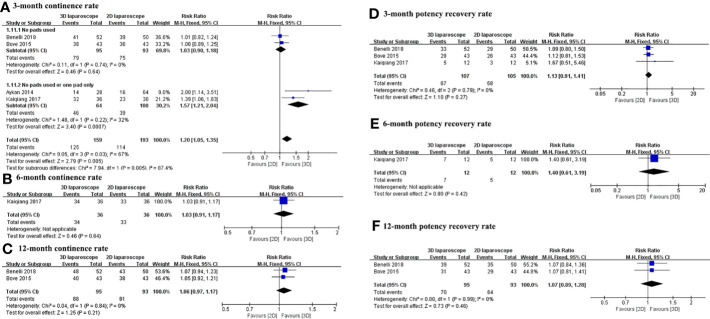
Forest plot comparing the functional outcomes of 3D and 2D LRP. Urinary continence rate at 3 **(A)**, 6 **(B)**, and 12 **(C)** months; potency recovery rate at 3 **(D)**, 6 **(E)**, and 12 **(F)** months.

The continence and potency recovery rates for 3D and 2D LRP are presented in **Table 4**. **Figure 4** depicts the pooled results of functional outcomes. The overall urinary continence results at 3, 6, and 12 months were available from four studies with a pooled RR of 1.20 (95% CI 1.05 to 1.35; *p*=0.0007), 1.03 (95% CI 0.91 to 1.17; *p*=0.64), and 1.06 (95% CI 0.97 to 1.17; *p*=0.21), respectively. The urinary continence rate at 3 months was higher for 3D LRP than for 2D LRP (**Figure 4A**). No difference was found between two groups regarding continence rate at 6 and 12 months (**Figures 4B, C**). Two studies defined patient’s continence as no use of any pads (14, 15), and other studies defined it as the use of 1 pad or less per day (16, 19).

**Table 4:** Urinary continence and potency recovery of the included studies.

Study	Surgical technique	Continence definition	Potency definition	Continence rate, n/N			Potency recovery rate, n/N		
				3 mo	6 mo	12 mo	3 mo	6 mo	12 mo
Aykan (16)	2D	0-1 pad	–	16/64	–	–	–	–	–
	3D			14/28					
Bove (14)	2D	no pads	IIEF-6 score ≥17	36/43	–	38/43	26/43	–	29/43
	3D			38/43		40/43	29/43		31/43
Kaiqiang (19)	2D	0-1 pad	IIEF-5 score ≥17	23/36	33/36	–	3/12	5/12	–
	3D			32/36	34/36		5/12	7/12	
Benelli (15)	2D	no pads	IIEF-5 score ≥16	39/50	–	43/50	29/50	–	35/50
	3D			41/52		48/52	33/52		39/52

There was no difference between groups in terms of potency recovery rate at 3, 6, and 12 months (**Figures 4D-F**). Three studies defined potency recovery as an IIEF-6 score≥17, IIEF-5 score≥16, and IIEF-5 score≥17, respectively (14, 15, 19).

### Heterogeneity

Moderate to high heterogeneity among studies was found for most of perioperative outcomes. Low heterogeneity was found in oncological and functional outcomes. However, estimating low heterogeneity for these outcomes should be cautious, because von Hippel PT has proven that I^2^ has a substantial bias when the number of included studies is too small (20). Furthermore, although there was heterogeneity among the included studies, this is not surprising given differences in surgical technique, medical equipment, and ethnicity.

### Assessment of publication bias

We were unable to assess publication bias because the testing ability was insufficient when there were 10 or fewer studies (21, 22).

## Discussion

With the assistance of 3D imaging systems, surgeons can perform complex laparoscopic surgeries with greater precision, flexibility, and effectiveness. Descazeaud et al. assumed that robotic-assisted radical prostatectomy (RARP) may become the standard technique for localized prostate cancer (23). However, there are regional differences in the use of robotic-assisted laparoscopic surgery compared to traditional laparoscopic surgery (24). In fact, RARP is only common in developed countries such as Europe and America, and most regions still rely on LRP as the primary surgical treatment for PCa. Due to the drawbacks such as inducing fatigue and damaging visual acuity in surgical practice, the adoption of 3D LRP has been slow in clinical settings. The latest advances in 3D laparoscopic technology have significantly improved the performance, precision, and hand-eye coordination of laparoscopic surgery, while providing greater depth perception during the surgical process and minimizing dizziness for surgeons (25, 26). Therefore, in recent years, there has been a resurgence in 3D laparoscopic surgery. According to a meta-analysis of general surgery procedures, it was determined that 3D laparoscopy provides superior surgical efficacy compared to 2D laparoscopy (27). To the best of our knowledge, no systematic reviews provide conclusive priority of 3D over 2D LRP in terms of their perioperative, oncologic, and functional outcomes. After considering all comparative studies, the present meta-analysis revealed that 3D LRP offers favorable outcomes compared with 2D LRP, including operative time, blood loss, days of drainage, and early continence. However, there was no conclusive evidence that 3D LRP was advantaged in terms of oncological and functional outcomes (except for continence rate at 3 months).

Our pooled analysis showed that operative time was lower in 3D LRP than those in 2D LRP. And no statistically significant difference was found between the two groups in terms of anastomosis time. A randomized comparative study indicated that 3D LRP may have limited advantages over 2D LRP only in terms of shortened operative time (13). However, in their study, Bove et al. (14), Benelli et al. (15), and Aykan et al (16) showed significantly shorter operative time and anastomosis time. Moreover, in Aykan et al.’s study, the variance in operation time and anastomosis time were almost perfectly aligned between the two groups (16). Authors speculated that the improvement in surgical duration could be primarily attributed to the facilitation of urethrovesical anastomosis. It was typically considered a most critical and time-consuming step in RP (28). With regard to blood loss, our research findings suggested that 3D LRP can significantly reduce blood loss. Lower blood loss may be attributed to magnified 3D view, which enables surgeons to better visualize vascular anatomy and perform more precise maneuvers (15, 16).

Hospital day was similar in 3D than 2D group. Due to variations in economic healthcare systems and healthcare insurance policies, hospitalization durations differ across different countries. In addition, the occurrence of postoperative complications, even mild ones, can significantly affect the length of hospital stay. A patient with urinary leakage due to anastomotic disruption caused by clot occlusion of an indwelling urinary catheter resulted in a hospitalization period approximately twice as long as other patients (17).

Concerning safety outcomes, there was no statistically significant difference between 3D and 2D group in terms of the overall complication rate. As is well known, postoperative complications are closely related to the safety of the surgery. Schmitges et al. compared the incidence of complications in minimally invasive radical prostatectomy between early (2001–2005) and late (2006–2007) study years and found that complication rates after surgery decreased over time (29). Authors analyzed that these observations may be associated with improved expertise and a higher proportion of sophisticate surgeons. Similarly, Bove et al. concluded that experience of the surgeon can also affect the complication rates to some extent (14).

Respecting oncological outcomes, PSM rate was similar between the two groups. In Bove et al.’s study (14), there was no significant difference in PSM rate between 3D and 2D LRP. On the contrary, when the cases were grouped by pathological stage, there was a significant difference in the rate of PSM occurrence between the two groups of patients with pT2c/pT3 disease. However, Huang et al. (30) conducted a meta-analysis that yielded results entirely contrary to those of Bove et al., despite the former’s comparison of RARP and LRP. It is undeniable that values of PSM rate in ≥pT3 tumors became higher than those in T2 tumors. Furthermore, PSM was also associated with surgeon’s experience, tumor stage, PSA level, and Gleason score.

With regard to BCR-free rates at 3, 6, and 12months, there was no statistically significant difference between 3D and 2D LRP group. PSM is associated with an increased likelihood of BCR and the need for adjuvant therapy (31). Yossepowitch et al. (32) reported that having positive margins is linked to a twofold increase in the risk of experiencing a biochemical relapse. In theory, tumors that are staged later should have a higher BCR rate owing to patients with a later stage of pathological staging have a higher PSM rate. Unfortunately, there is currently no study comparing BCR in pT2c and pT3 tumors in the included studies. Therefore, more studies are needed to clarify the relationship between BCR and tumor stage.

The recovery of urinary continence is the most important factor affecting the quality of life after radical prostatectomy (33), and it is also the most concerning issue for patients. The concept of continence is not always consistent. In one study defining continence as the use of 1 pad or less per day, Aykan et al. (16) concluded that 3D LRP was associated with higher early continence rates in comparison with 2D LRP. However, in another more recent RCT, Bove et al. (14) defined continence as no use of any pads and reported that no statistically significant difference was found in terms of overall continence rate. In our meta-analysis, the urinary continence rate at 3 months was higher for 3D LRP than for 2D LRP. The pooled RR for urinary continence rate at 6 and 12 months were 1.03 (95% CI 0.91 to 1.17; *p*=0.64) and 1.06 (95% CI 0.97 to 1.17; *p*=0.21), respectively. Although them did not reach a statistically significant difference, the trend is favorable to the 3D LRP group.

Similarly, there was no conclusive evidence that 3D LRP was advantaged in terms of the recovery of potency. However, the recovery of sexual dysfunctions after RP is a difficult outcome to evaluate and compare. Having a comprehensive discussion with the patients about preoperative erectile function, the actual incidence of postoperative erectile dysfunction (ED), and the concept of drug-assisted or spontaneous erection is a key issue in understanding ED prevention and promoting satisfactory erectile function recovery after RP (34). When comparing the functional outcomes of 2D and 3D LRP, it is necessary to pay attention to some key issues. First, we can come up with different outcomes when using different method to assess continence and potency. Second, the recovery of EF is influenced by many factors, including the patient’s age, preservation of sexual nerves, type of surgery, surgical techniques, and surgeon surgical skill (34). Third, collecting functional outcome data through a questionnaire survey may lead to errors in the study. Patients may not fully understand the questions or be unwilling to answer, which may result in inaccurate data. Overall, caution should be exercised when evaluating and comparing functional outcomes between 2D and 3D LRP.

This review has several limitations. First, this study was the lack of data on oncological and functional outcomes. The results showed that there were no statistically significant differences between 3D and 2D LRP group concerning oncologic and functional outcomes. However, these results were based on a limited number of studies and need to be confirmed in future studies. Second, studies included in this meta-analysis were retrospective or prospective cohort studies, which may have inherent biases. Third, the number of surgeons and their level of experience in surgery are not comparable between the included studies.

## Conclusion

In conclusion, 3D LRP showed some advantages over 2D LRP in terms of perioperative outcomes, but there were no significant differences in oncologic and functional outcomes (except for continence rate at 3 months). However, due to the limited number and quality of the included studies, further studies are needed to validate these findings.

## Data availability statement

The original contributions presented in the study are included in the article/supplementary material. Further inquiries can be directed to the corresponding author.

## Author contributions

TW had full access to all the data in the study and takes responsibility for the integrity of the data and the accuracy of the data analysis. Study concept and design: TW and HS. Acquisition of data: HS and XD. Analysis and interpretation of data: XD. Drafting of the manuscript: HS and TW. Critical revision of the manuscript for important intellectual content: HS, XD, and TW. Statistical analysis: HS. Supervision: TW. All authors contributed to the article and approved the submitted version.
